# Measuring Nepotism through Shared Last Names: Are We Really Moving from Opinions to Facts?

**DOI:** 10.1371/journal.pone.0043574

**Published:** 2012-08-24

**Authors:** Fabio Ferlazzo, Stefano Sdoia

**Affiliations:** Department of Psychology, Sapienza University of Rome, Rome, Italy; Philipps-University Marburg, Germany

## Abstract

Nepotistic practices are detrimental for academia. An analysis of shared last names among academics was recently proposed to measure the diffusion of nepotism, the results of which have had a huge resonance. This method was thus proposed to orient the decisions of policy makers concerning cuts and funding. Because of the social relevance of this issue, the validity of this method must be assessed. Thus, we compared results from an analysis of Italian and United Kingdom academic last names, and of Italian last and given names. The results strongly suggest that the analysis of shared last names is not a measure of nepotism, as it is largely affected by social capital, professional networking and demographic effects, whose contribution is difficult to assess. Thus, the analysis of shared last names is not useful for guiding research policy.

## Introduction

In a very recent paper, Allesina [1] proposed a method to measure the diffusion of nepotism in Italian academia through an analysis of shared last names. The rationale was that if in any organization (for instance, a discipline in academia) the number of shared last names is higher than expected by chance, members of the same families are over-represented in that organization, hence nepotism. Because in Italy children take the last name of their father, this method is supposed to measure father-progeny and inter-sibling nepotism, not mother-progeny and nuptial nepotism. It is worth noting that nuptial nepotism is especially difficult to assess, on a statistical basis, not only because in many countries spouses keep their birth names and there is a growing tendency of couples not to marry, but also because in some countries (e.g., USA, Canada, Germany, Switzerland) dual-career supporting programs have been established since many years, albeit dual-career hiring is still a controversial issue [2]. In Italy, according to a recently approved legislative act, universities cannot employ close relatives, including spouses, in the same department.

Results reported in [1] were interpreted as demonstrating a diffuse and pervasive use of nepotistic practices in acquiring academic positions in Italy. It was also suggested that the method should be used by policy makers to target cuts and funding to prevent this phenomenon from devastating the Italian university system.

These conclusions have had an immediate and widespread resonance in Italy and abroad on mass media and other national and international venues [3]. This is not surprising because allegations of nepotism always attract considerable attention. Indeed, nepotism is inherently contrary to any idea of equality and fairness. Interestingly, evidence of nepotism is usually limited to single blatant cases, rumours, suspects, and sometimes stereotypes, but data about the real diffusion of nepotism are usually lacking. Again, this is not surprising because measuring nepotism is difficult. Indeed, nepotism refers to hiring someone who is undeserving, because of family ties. Thus, measuring the diffusion of nepotism requires a review of the merits of all the individuals involved, which is clearly an impossible mission. This is one of the reasons why the method Allesina proposed is of such interest. Furthermore, the analysis of shared last names as it was proposed in [1] does not require knowing the frequency of last names in the general population. Indeed, the analysis of shared last names had already been proposed and used to measure nepotism [4]; however, the frequency of last names in the general population was taken into account. As Allesina [1] argued, the approach followed in [4] is likely more accurate, but estimating the frequency of names in a population may be difficult. For example, complete and reliable sources of names such as the population registries that exist in most countries are not open to the public consultation for reasons of privacy. Other sources such as those commonly found on the web are either based upon proxies such as phone books, thus representing a subset of the population, or do not specify their data gathering system, thus arising a reliability issue. Instead, the method proposed in [1] is relatively simple, being based on only the last names of academics.

However, we show herein that the method does not measure nepotism. This is a key point, as the validity of any method intended to measure something should be demonstrated.

In addition to nepotism, other factors could account for the high proportion of shared names in some scientific disciplines, even if shared names were perfect indicators of family ties, which is obviously not true. Indeed, the phenomenon of parents’ career following has been observed and studied in many fields, such as politics [5], [6], [7], medicine [8], law [9], business [10], and sports [11]. For example, in Canada and Denmark up to 6% of men have the same employer as their fathers, and this is positively related to paternal earnings [12], [13]. Indeed, the intergenerational transmission of economic status is the focus of a growing literature in economics [14].

There are many reasons why children may decide to enter the same career paths as their parents, including physical-capital transfer, human-capital transfer, brand-name loyalty transfer, and of course nepotism [11]. The notion of social capital has been frequently used in these cases. Social capital can be roughly defined as the goodwill that is engendered by social relationships and can be used to facilitate action [15], and how social relationships promote the acquisition of skills and traits valued in the job market [16]. A large number of studies have demonstrated the relevance of social capital and professional networks for career pursuing [17], [18]. For example, data from the National Longitudinal Survey of Youth (NLSY) showed [18] that informal contacts were the most frequent source for 1982 jobs. Such contacts were used by roughly 50% of each race/gender group. Because social capital and professional networks are so important, the family of origin may be an important asset for being successful in pursuing a career.

Whereas no specific study exists about occupation following in academic career, it is well known that social capital and professional networking play a relevant role in getting a job in academia worldwide. Of note, networking not only serves as a source of information about job openings, but also for emotional support, suggestions, skills development, and problem-solving. Thus, it is likely that even in academia an advantage exists in capitalizing upon the social capital and professional network from the family of origin. These types of advantages enjoyed by the children of academics in pursuing an academic position may be socially disturbing, but they are not nepotism, and countermeasures should aim at filling the gaps rather than at punishing those who benefit. Interestingly, social capital as a determinant of the proportion of shared last names in academia was discarded in [1] as very unlikely, albeit without offering empirical evidence.

The significantly higher than expected by chance proportion of shared names in some disciplines in Italian academia may also be due to more trivial, demographic reasons. Indeed, a clear North-South trend in Italy was reported, with the likelihood of nepotism (actually, proportion of shared names) being greater in the South compared to the North. It is worth noting that Italy has witnessed strong South-North migration flows [19]. As last names in Italy as in other countries have a regional origin, such migration might have produced a larger variability of last names in the northern regions and a lower variability (i.e., more shared names) in the southern regions. Together with the uneven distribution of research centres across regions in Italy, demographic factors might account for the higher proportions of shared names in some disciplines.

As results of the analysis of shared last names could be due to factors different from nepotism, and because the strong claim that the method should be used to orient policy makers, validating the method as a tool to measure nepotism is paramount.

To investigate this issue, we applied the same procedure to a dataset of academics in the UK. It is commonly held that academic recruitment in the UK is based on individual merits and accomplishments, and that nepotism only has a very marginal role, if any. Also, recruitment for academic positions in the UK takes place essentially at the department or institution level, without much involvement of academics in the same or related disciplines outside the institution that opens the call. Thus, any sign of nepotism should be expected to occur for institutions, but should fade out for disciplines. Finally, the dataset of academics we used was composed of researchers who were selected for the 2008 Research Assessment Exercise (RAE). Such a selection was likely performed to boost the probability of a positive evaluation. Because nepotism refers to hiring someone regardless their merits, such a selection should reduce any sign of nepotism in the dataset. Thus, if the method proposed in [1] really measures nepotism, we should expect no significant differences between the observed and expected numbers of distinct last names within disciplines in the UK.

## Results

We first replicated the study in [1] using the same method, algorithm, and approximately the same dataset of Italian academics. The dataset includes 61,730 records (all the academics in Italy on 31 December 2009) and 27,307 unique last names.

The same Monte Carlo simulation was used to compute the approximate p-value for each discipline (macro-sector) measuring the probability of finding a smaller number of distinct names in 10^5^ random drawings without replacement from the whole dataset. As expected, the results were virtually identical to the results originally reported in [1]. Indeed, it was found that the number of distinct last names was significantly (p<.05) less than expected in 9 of 28 macro-sectors (32.14%; Table 1). Especially, Medical Sciences and Law showed the largest difference between the observed and expected number of distinct last names. According to the rationale underlying the method, these are fields with a high probability of nepotism. It is worth noting that because the 9 macro-sectors with a significantly smaller than expected number of distinct names encompass the majority of Italian academics (approximately 51%), it was concluded that nepotism is prominent in Italian academia. This seems to be a bold claim because that cases of nepotism exist in a discipline does not necessarily mean that all of the academics in that discipline are involved. In fact, the overall number of missing names relative to the expected values was 738.6 (from the analysis on macro-sectors, 312 in the Medical Sciences) in the Allesina’s study and 755.0 in the current study. Of >27,000 last names (for >60,000 academics), that these figures suggest prominent nepotism is at least debatable.

**Table 1:** Likelihood of nepotism for discipline in Italy.

Disciplines in Italy	People	Last names	Expected	p-value	Given names	Expected	p-value
Medical Sciences	10946	7547	7874.4	**<.001**	2030	2059.1	0.173
Law	5124	4013	4192.7	**<.001**	1208	1229.6	0.186
Industrial Engineering	3172	2688	2752.7	**<.001**	760	895.5	**<.001**
Geography	383	365	374.0	**0.006**	221	220.0	0.573
Agriculture	2360	2070	2107.8	**0.008**	723	738.0	0.208
Education	676	636	649.7	**0.008**	348	324.8	0.985
Chemistry	3180	2717	2758.9	**0.015**	866	897.0	0.063
Mathematics	2551	2227	2262.4	**0.016**	752	776.4	0.095
Civil Engineering	3831	3209	3254.6	**0.019**	972	1013.9	**0.025**
Statistics	1215	1123	1137.5	0.058	466	478.7	0.187
History	1477	1350	1366.7	0.060	559	543.8	0.853
Earth Sciences	1202	1112	1126.0	0.062	441	475.3	**0.006**
Philology	1809	1636	1650.8	0.122	654	620.6	0.982
Political Sciences	1794	1625	1638.0	0.152	646	617.2	0.966
Veterinary	862	814	820.7	0.170	378	381.9	0.389
Informatics	837	792	797.9	0.193	356	374.5	0.063
Life Sciences	5212	4233	4254.5	0.216	1253	1244.0	0.657
Physics	2499	2209	2220.4	0.243	698	766.2	**<.001**
Economics	3764	3193	3204.4	0.303	946	1002.2	**0.004**
Electronic Engineering	2068	1862	1867.7	0.348	513	677.1	**<.001**
Art History	827	787	788.7	0.413	382	371.5	0.823
Archaeology	714	685	684.9	0.535	357	336.9	0.968
Sports-Related Studies	134	133	132.8	0.680	96	100.7	0.185
Near Eastern Studies	315	310	308.9	0.729	205	191.6	0.967
Psychology	1246	1171	1164.9	0.768	537	486.7	>.999
Anthropology	200	199	197.4	0.918	151	137.2	0.991
Linguistics	2194	2017	1971.8	0.999	952	703.7	>.999
**Philosophy**	1138	1119	1069.2	>.999	450	458.6	0.276

Results of the Monte Carlo simulation for each discipline (sector) from the Italian dataset of academics. The number of academics in the discipline (People), the number of distinct last and given names (Last names, Given names), the expected number of last and given names (Expected), and the associated p-value, measuring how probable it is to find an equal or lower number of names by chance, are reported. Significant *p*-values are in bold.

We then applied the same procedure to the dataset of academics in the UK who were selected for the 2008 RAE. For each researcher, the 2008 RAE output includes the name, affiliation, and discipline (Unit of Assessment - UoA; Table S1). The dataset includes 62,157 records containing 26,615 unique last names. To better compare the Italian and UK results, some of the UoAs have been collapsed into broad disciplines similar to those defined in Italy (Table S2).

Unexpectedly, the results were worse than the results from the Italian dataset (Table 2). Indeed, the number of distinct last names was significantly (p<.05) less than expected by chance in 15 of 33 disciplines (45.45%). These fields included 33,500 researchers. Hence, similar to Italy, the majority of UK academics (53.9%) work in disciplines that display a smaller number of last names than expected by chance. Medical Sciences and Life Sciences had the largest difference between the observed and expected number of distinct last names. The number of disciplines showing a significant excess of shared names was not determined by the removal of duplicated records (see Materials and Methods), as the proportion of removed duplicates does not change between the disciplines with and without an excess of shared names (Z = −1.55, p = .13). These results strongly refute the conclusions that the smaller than expected number of distinct last names is an indicator of nepotism in academia, and that the method should be used for guiding research policy. Indeed, as nepotism effects are unlikely in the present UK dataset, that an even larger number of disciplines, compared to the Italian dataset, shows a significantly smaller than expected number of distinct names argues against such a conclusion. More likely, factors related to the social capital and professional network effects in academic jobs, or to the distribution of the last names in a specific population are involved. To check this last hypothesis, we applied the same method to the given names of the Italian academics. It is clear that nepotism cannot have an effect on the number of distinct given names in any discipline. Also, other family-related factors do not determine how parents choose the given names of their children. Thus, we should expect no difference between the observed and expected number of distinct given names in Italian academia. Of 61,730 researchers, there were 7147 unique given names. The results of the same Monte Carlo simulation were somewhat surprising. Indeed, the number of distinct given names was significantly (p<.05) less than expected by chance in 6 of 28 disciplines (Table 1). These fields included 16,536 researchers. Hence, following the rationale proposed in [1], a large number of academics in Italy (26.8%) work in fields wherein a bias toward hiring people with certain given names exists, which is quite unlikely, even in Italy. Obviously, these findings also stand against the idea that nepotism can be measured as the frequency of shared names. Indeed, nepotism (or any other kind of intergenerational transmission of academic job) does not affect how academics name their children. Instead, in Italy given names have a regional distribution, though less marked than last names, and different disciplines have different composition in terms of gender or age prevalence. Thus, it can be concluded that statistical features of the distribution of last names affect whether or not a discipline shows a significantly lower than expected frequency of shared names.

**Table 2:** Likelihood of nepotism for discipline in UK.

Disciplines in the UK	People	LastNames	Expected	p-value
Pharmacy	550	484	512.5	**<.001**
Geography	1417	1128	1227.3	**<.001**
Archaeology	652	570	601.4	**<.001**
Political Sciences	4461	3229	3344.1	**<.001**
Architecture	1327	1106	1156.8	**<.001**
Agriculture	1204	989	1059.0	**<.001**
Earth Sciences	1425	1172	1233.5	**<.001**
Chemistry	1337	1074	1164.6	**<.001**
Education	2038	1595	1697.7	**<.001**
English Language and Literature	2128	1646	1763.7	**<.001**
Medical Sciences	9884	6045	6463.8	**<.001**
Life Sciences	3591	2649	2779.9	**<.001**
History	2018	1638	1683.0	**0.004**
Electronic Engineering	946	834	849.4	**0.006**
Sports-Related Studies	522	477	487.9	**0.043**
Industrial Engineering	3399	2617	2651.8	0.072
Business and Management Studies	3870	2926	2963.4	0.074
Classics, Ancient History, Byzantine and Modern Greek Studies	523	480	488.8	0.086
Civil Engineering	583	532	541.5	0.086
Anthropology	435	405	410.5	0.162
Library and Information Management	337	319	321.6	0.285
Philosophy	1256	1094	1100.5	0.307
Law	1913	1598	1605.2	0.338
Linguistics	367	348	349.0	0.439
Physics	2016	1680	1681.5	0.474
Psychology	1865	1570	1569.4	0.526
Statistics	445	425	419.4	0.920
American, Middle Eastern, African, Asian, European, Celtic Studies	1124	1012	994.8	0.947
Philology	1263	1128	1106.0	0.971
Mathematics	1786	1567	1510.2	>.999
Economics	1270	1155	1111.6	>.999
Informatics	2059	1786	1713.2	>.999
**Art History**	4146	3244	3142.5	>.999

Results of the Monte Carlo simulation for each discipline (sector) from the UK dataset of academics. The number of academics in the discipline (People), the number of distinct last names (Last names), the expected number of last names (Expected), and the associated p-value, measuring how probable it is to find an equal or lower number of distinct last names by chance, are reported. Significant *p*-values are in bold.

Furthermore, we also used a logistic regression model to assess the effect of geography and institution on the probability that two UK academics share their last name. Results of the logistic regression (Table S3) were similar to those obtained in Italy [1], showing that distance has a significant effect in 14 disciplines, wherein the smaller is the distance the higher is the probability that two researchers have the same last name. The institution also has a significant effect, in addition to the distance, in 8 disciplines, wherein the probability of two researchers have the same last name is boosted when they belong to the same institution. However, it should be noted that the geographic clustering of last names in the UK is likely very different from that in Italy. Indeed, the pattern of internal migration in the UK was mostly characterized by short moves, and only since the second half of the 20th century by a North to South net flow [20]. Furthermore, academic institutions in the UK are less evenly distributed across the country than in Italy, being concentrated in the South (England, especially the London area). This also makes it difficult to fully compare the results from the logistic regressions in Italy and in the UK.

Finally, we computed the relative frequency of name-sharing within institutions in the UK. Results show in the UK the same range of within-institution frequencies of pairs of academics sharing their last name that was reported in Italy, and the same distribution across institutions (Figure S1).

## Discussion

Everybody agrees that nepotistic practices are detrimental for academia. Analysis of shared last names among academics has been proposed to measure the diffusion of nepotism, the results of which have had huge resonance. This method was also proposed to orient the decisions of policy makers concerning cuts and funding. Whereas the feasibility of this last proposal may be debatable (e.g., nepotism is illegal, at least in Italy, and thus allegations of nepotism should be proved on a factual, not statistical, basis), the method could still be used to draw a general picture of the phenomenon. Though, because of the social relevance of this issue, the validity of this method must be assessed. Our results suggest that the analysis of shared names should not be used as a tool to measure the diffusion of nepotism in academia or in any organization. Indeed, social capital factors are likely the most important determinants of the proportion of shared last names in academia, as shown by the strictly similar results from the analysis of last names amongst academics in Italy and the UK. Also, demographic factors play a role as well, as shown by the significantly smaller than expected number of distinct given names of academics in Italy. Our results do not imply that nepotism does not exist in Italian academia, for it surely is. However, the results do show that the analysis of shared last names as it was proposed in [1] is not a valid method to measure how diffuse nepotism is. It might be possible that including the frequency of last names in the general population, such as in [4], yields more reliable indices of nepotism, but that should be demonstrated. Indeed, it is worth noting that our results strongly suggest that any method to measure nepotism should be carefully validated, for example by comparing different countries. This is especially true when methods are proposed to be used for making decisions, as decisions are often important and do have consequences, even in academia.

## Materials and Methods

### Monte Carlo Simulation

Allesina [1] proposed an index for measuring the diffusion of nepotism in any organization, including scientific disciplines in academia. If a discipline includes *K* individuals and *N* distinct last names, then the probability that observing *N* or less distinct last names is due to chance is estimated by a Monte Carlo simulation. For each discipline, a large number of samples are created by drawing at random without replacement *K* individuals from the entire dataset. For each sample, the number, *N’*, of distinct last names is computed, and the frequency distribution of *N’* across the samples yields the probability of observing a number of distinct names, *N’≥N*, by chance. If the probability is <0.05, it can be concluded that the observed number of distinct last names in the original sample was not due to chance but to nepotism. As in the present study a large number of tests was run, controlling for the alpha inflation was needed. As in [1], we controlled the proportion of type I errors among all rejected null hypotheses by setting the False Discovery Rate (FDR) to.05. The FDR was estimated through the procedure described in [21]. The bootstrap procedure was used to estimate the π0 parameter [22] (for a general view on the bootstrap procedures, see [23], [24]). In our results, the 0.05 level of significance corresponded to an FDR <0.05.

The expected number of distinct last names under the hypothesis of chance is the average of the numbers of distinct last names in the samples. We programmed the Monte Carlo simulation in C using the function *gsl_ran_choose* from the GNU Scientific Library 1.14 (www.gnu.org/gsl). For each discipline, the simulation was run on 10^5^ random samples.

### Logistic Regression

We used logistic regression to predict for each pair of academics if they share the last name based on how geographically close they are, and on whether they work in the same institution, according to the following model:
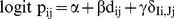
where I_i_ and I_j_ are the institutions the academics i and j belong to, d_ij_ is the distance between their institutions, δ*_Ii_,_Jj_* is a Kronecker delta that takes value 1 when I_i_ = I_j_ and 0 otherwise. Negative *β* stands for an increase in probability of sharing their last name the geographically closer the two academics are. Positive γ stands for an increase in probability when the two academics work in the same institution. Distances were computed through the geographic coordinates of the UK institutions included in the 2008 RAE as derived from their postal codes. The British Institute in Paris was excluded from the analysis, as it is located outside the UK. The University Marine Biological Station (Millport) was also excluded because it is an institute of the University of London though it is located in Scotland.

### Italian Dataset

The Italian dataset used herein was downloaded from the Ministry of Education website (http://cercauniversita.cineca.it) and refers to the academics as of 31 December 2009, whereas the dataset used in [1] was downloaded on 8 October 2010. As data about academics for the past years refer to the situation at the end of each year, the present dataset was slightly different from that used in [1], which is no longer available. We preferred the 2009 dataset over the 2010 dataset because academics in Italy often retire (and get hired) during March and November each year. Thus, the 2010 dataset does not include the academics who retired in November 2010, but were still active on 8 October 2010.

The dataset includes the given and last names, institution, department, and discipline of 61,730 academics in Italy (all of the academics in Italy). The number of unique last names was 27,307, of which 3 were shared by ≥100 people (Rossi, 228; Russo, 152; and Ferrari, 112). A total of 17,289 names were associated with only 1 academic, 4626 names were shared by 2 researchers, and 5392 names were shared by ≥3 academics.

In Italy, each academic is associated with one of 370 disciplinary sectors of interest, as defined by the Ministry of Education, and clustered into 28 macro-sectors. For instance, the Psychology macro-sector (M-PSI) is composed of 8 micro-sectors (e.g., General Psychology, M-PSI/01). The list of macro-sectors is reported in Table S2. There were 26,238 assistant professors, 17,594 associate professors, and 17,908 full professors. Because we did not take into consideration geographic variables, we did not remove academics appointed at distance learning universities.

### United Kingdom Dataset

Unfortunately, there is no database of academics in the UK (or in other countries in Europe) that is complete and open to public consultation, as in Italy. Thus, we used the dataset of the UK academics resulting from the RAE output. Higher education institutions in the UK go periodically through an exercise undertaken on behalf of four UK higher education funding councils to evaluate the quality of their research. The results of the last exercise (2008) are available to the public on the 2008 RAE website (http://www.rae.ac.uk), and include the last name, initials, institution, and discipline (UoA, Table S1) of UK academics who were selected by their institutions for submitting their research products. The selection of academics was likely performed to boost the probability that the institution receives a positive evaluation. As nepotism refers to hiring someone regardless their merits, such a selection should reduce any sign of nepotism in the dataset. Furthermore, the UoA researchers were associated with were decided, by themselves and the institutions, on the basis of their research activity, similar to what happens in Italy.

Given the procedures for submitting to the RAE, though, individuals that moved from one institution to another during the period of the exercise may give rise to multiple entries if they were selected by more than one institution. As only the initials of the given names were recorded, it was impossible to distinguish duplicates. Two records with the same last name and initials may refer to two different individuals or to a duplicate. In order to avoid the presence of duplicates in the dataset, we clustered the UoAs into three broad groups, as follows: medical sciences (UoAs 1–12), science (UoAs 13–29), and humanities (UoAs 30–67). If two or more records with the same name and initials referred to UoAs in the same group, they were considered to belong to the same individual, and duplicates were removed; if the records referred to UoAs in different groups, they were considered to belong to different individuals and retained. This procedure makes it very likely that duplicates are removed from the dataset, but it also makes it probable that different individuals with the same name and initials get removed as well. However, this also reduces the number of people sharing the same name, and hence the level of “nepotism” in the dataset. Originally, the number of duplicated records (same last name and initials of a record already present in the dataset) was 9158. Using the algorithm described above, 6405 records were removed. The final dataset included 62,157 records from 159 higher education institutions across the UK. There were 26,615 unique last names, of which 19,331 names were associated with only 1 academic, 3380 names were shared by 2 researchers, and 3904 were shared by ≥3 academics.

To better compare the Italian and UK results, some of the UoAs have been collapsed into broad disciplines similar to those defined in Italy (Table S2).

As the present study only involved analyses on public domain data, and identification of individual researchers was impossible, no ethics committee approval was required.

## Supporting Information

Figure S1 Distribution of the frequencies of same name pairs (*1000) within institutions in Italy and in the UK.
(PDF)

Table S1 **Units of Assessment (disciplines) as defined for the 2008 Research Assessment Exercise in the United Kingdom.**
(PDF)

Table S2 **Macro-sectors (disciplines) as defined by the Italian Ministry of University, and the correspondent Units of Assessment as defined for the 2008 Research Assessment Exercise in the United Kingdom.**
(PDF)

Table S3 **Results of the logistic regression.** For each coefficient, the fitted value and the associated probability are reported. á is the intercept, â the coefficient accounting for geographic distance and ã the coefficient accounting for the effect of belonging to the same institution.
(PDF)
