# Supplementary material for: Measuring Nepotism through Shared Last Names: Are We Really Moving from Opinions to Facts?
Source: PLoS One. 2012 Aug 24;7(8):e43574. doi: 10.1371/journal.pone.0043574 (PMC3427342; doi:10.1371/journal.pone.0043574)

Figure S1. Distribution of the frequencies of same name pairs (*1000) within institutions in Italy and in the UK.

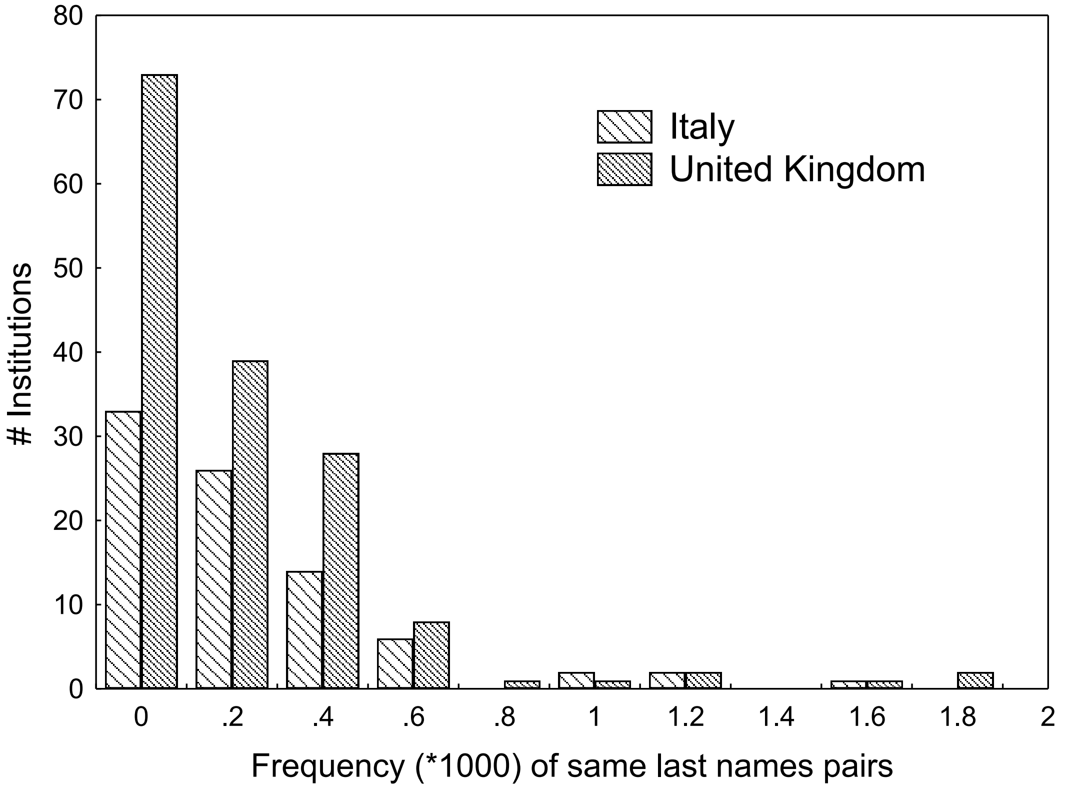

Supplement: Figure S1 — Distribution of the frequencies of same name pairs (*1000) within institutions in Italy and in the UK. (PDF) [file pone.0043574.s001.pdf]
