# Supplementary material for: Measuring Nepotism through Shared Last Names: Are We Really Moving from Opinions to Facts?
Source: PLoS One. 2012 Aug 24;7(8):e43574. doi: 10.1371/journal.pone.0043574 (PMC3427342; doi:10.1371/journal.pone.0043574)
Supplement: Table S1 — Units of Assessment (disciplines) as defined for the 2008 Research Assessment Exercise in the United Kingdom. (PDF) [file pone.0043574.s002.pdf]

Table S1. Units of Assessment (disciplines) as defined for the 2008 Research Assessment Exercise in the United Kingdom.

**Unit of Assessment and description**	**Unit of Assessment and description**
1. Cardiovascular Medicine	35. Accounting and Finance
2. Cancer Studies	36. Business and Management Studies
3. Infection and Immunology	37. Library and Information Management
4. Other Hospital Based Clinical Subjects	38. Law
5. Other Laboratory Based Clinical Subjects	39. Politics and International Studies
6. Epidemiology and Public Health	40. Social Work and Social Policy & Administration
7. Health Services Research	41. Sociology
8. Primary Care and Other Community Based Clinical Subjects	42. Anthropology
9. Psychiatry, Neuroscience and Clinical Psychology	43. Development Studies
10. Dentistry	44. Psychology
11. Nursing and Midwifery	45. Education
12. Allied Health Professions and Studies	46. Sports-Related Studies
13. Pharmacy	47. American Studies and Anglophone Area Studies
14. Biological Sciences	48. Middle Eastern and African Studies
15. Pre-clinical and Human Biological Sciences	49. Asian Studies
16. Agriculture, Veterinary and Food Science	50. European Studies
17. Earth Systems and Environmental Sciences	51. Russian, Slavonic and East European Languages
18. Chemistry	52. French
19. Physics	53. German, Dutch and Scandinavian Languages
20. Pure Mathematics	54. Italian
21. Applied Mathematics	55. Iberian and Latin American Languages
22. Statistics and Operational Research	56. Celtic Studies
23. Computer Science and Informatics	57. English Language and Literature
24. Electrical and Electronic Engineering	58. Linguistics
25. General Engineering and Mineral & Mining Engineering	59. Classics, Ancient History, Byzantine and Modern Greek Studies
26. Chemical Engineering	60. Philosophy
27. Civil Engineering	61. Theology, Divinity and Religious Studies
28. Mechanical, Aeronautical and Manufacturing Engineering	62. History
29. Metallurgy and Materials	63. Art and Design
30. Architecture and the Built Environment	64. History of Art, Architecture and Design
31. Town and Country Planning	65. Drama, Dance and Performing Arts
32. Geography and Environmental Studies	66. Communication, Cultural and Media Studies
33. Archaeology	67. Music
34. Economics and Econometrics	
