# Supplementary material for: Measuring Nepotism through Shared Last Names: Are We Really Moving from Opinions to Facts?
Source: PLoS One. 2012 Aug 24;7(8):e43574. doi: 10.1371/journal.pone.0043574 (PMC3427342; doi:10.1371/journal.pone.0043574)
Supplement: Table S2 — Macro-sectors (disciplines) as defined by the Italian Ministry of University, and the correspondent Units of Assessment as defined for the 2008 Research Assessment Exercise in the United Kingdom. (PDF) [file pone.0043574.s003.pdf]

Table S2. Macro-sectors (disciplines) as defined by the Italian Ministry of University, and the correspondent Units of Assessment as defined for the 2008 Research Assessment Exercise in the United Kingdom.

**Discipline**	**Macro-sector acronym (Italy)**	**Unit of Assessment (UK)**
Agriculture	AGR	16
American, Middle Eastern, African, Asian, European, Celtic Studies		47 to 50, 56
Archeology	L-ANT	33
Architecture		30, 31
Art history	L-ART	63 to 67
Business and management studies		36
Chemistry	CHIM	18
Civil engineering	ICAR	27
Classics, ancient history, Byzantine and modern Greek studies		59
Demography & ethnology	M-DEA	42
Earth sciences	GEO	17
Economics	SECS-P	34, 35
Electronic engineering	ING-INF	24
English language and literature		57
Geography	M-GGR	32
History	M-STO	62
Industrial engineering	ING-IND	25, 26, 28, 29
Informatics	INF	23
Law	IUS	38
Library and information management		37
Life sciences	BIO	14,15
Linguistics	L-LIN	58
Mathematics	MAT	20, 21
Medical sciences	MED	1 to 12
Near eastern studies	L-OR	
Pedagogy	M-PED	45
Pharmacy		13
Philology	L-FIL-LET	51 to 56
Philosophy	M-FIL	60, 61
Physical education	M-EDF	46
Physics	FIS	19
Political sciences	SPS	39 to 41, 43
Psychology	M-PSI	44
Statistics	SECS-S	22
Veterinary	VET	
