# Supplementary material for: Measuring Nepotism through Shared Last Names: Are We Really Moving from Opinions to Facts?
Source: PLoS One. 2012 Aug 24;7(8):e43574. doi: 10.1371/journal.pone.0043574 (PMC3427342; doi:10.1371/journal.pone.0043574)
Supplement: Table S3 — Results of the logistic regression. For each coefficient, the fitted value and the associated probability are reported. á is the intercept, â the coefficient accounting for geographic distance and ã the coefficient accounting for the effect of belonging to the same institution. (PDF) [file pone.0043574.s004.pdf]

Table S3. Results of the logistic regression. For each coefficient, the fitted value and the associated probability are reported. α is the intercept, β the coefficient accounting for geographic distance and γ the coefficient accounting for the effect of belonging to the same institution.

Discipline	α	p(α))	β	p(β))	γ	p((γ))
Pharmacy	-7.154	0.000	-0.00073	0.294	0.274	0.421
Agriculture	-7.263	0.000	-0.00068	0.028	-0.076	0.718
Earth sciences	-7.507	0.000	-0.00050	0.076	0.042	0.859
Chemistry	-7.341	0.000	-0.00009	0.002	0.535	0.001
Physics	-7.876	0.000	-0.00052	0.052	-0.018	0.926
Statistics	-8.025	0.000	-0.00117	0.438	-0.303	0.777
Informatics	-8.471	0.000	-0.00178	0.937	0.701	0.008
Civil engineering	-8.190	0.000	0.00011	0.903	0.476	0.415
Geography	-7.355	0.000	-0.00053	0.049	0.232	0.318
Archaeology	-7.395	0.000	-0.00062	0.394	-0.027	0.949
Business and management studies	-7.965	0.000	-0.00049	0.001	0.245	0.067
Library and information management	-8.509	0.000	0.00187	0.211	0.869	0.298
Law	-7.935	0.000	-0.00092	0.001	0.416	0.057
Demography & ethnology	-7.866	0.000	-0.00066	0.520	0.496	0.391
Psychology	-8.195	0.000	-0.00023	0.439	0.782	0.002
Pedagogy	-7.577	0.000	-0.00125	0.000	-0.223	0.243
Physical education	-7.610	0.000	-0.00173	0.176	0.689	0.198
English language and literature	-7.911	0.000	-0.00027	0.276	0.340	0.146
Linguistics	-7.598	0.000	-0.00134	0.350	-0.668	0.533
Classics, ancient history, Byzantine and modern Greek studies	-8.423	0.000	0.00138	0.156	1.586	0.000
History	-7.957	0.000	-0.00018	0.461	0.465	0.029
Medical sciences	-7.788	0.000	-0.00009	0.000	0.061	0.131
Life sciences	-7.658	0.000	-0.00002	0.184	0.101	0.279
Mathematics	-8.438	0.000	-0.00003	0.545	0.407	0.103
Electronic engineering	-8.153	0.000	-0.00061	0.001	0.455	0.006
Architecture	-7.833	0.000	0.00056	0.072	-0.210	0.545
Economics	-8.197	0.000	-0.00122	0.011	0.066	0.860
Philosophy	-7.983	0.000	-0.00087	0.041	0.236	0.436
Industrial engineering	-7.938	0.000	-0.00048	0.003	0.341	0.005
Political sciences	-8.046	0.000	-0.00011	0.352	0.199	0.105
American, Middle Eastern, African, Asian, European, Celtic Studies	-8.234	0.000	-0.00047	0.398	0.508	0.147
Philology	-8.104	0.000	-0.00076	0.099	1.277	0.031
Art history	-8.399	0.000	-0.00056	0.000	0.167	0.331
